# Local–Migrant Interaction in Everyday Life in an Ancient Tourism Town

**DOI:** 10.3390/ijerph17010266

**Published:** 2019-12-30

**Authors:** Hongxia Zhang, Qin Su, Guiqiang Qiao, Yingmei Yin, Xiaoxiao Wu, Wujie Xie

**Affiliations:** 1 School of Geography and Tourism, Anhui Normal University, Wuhu 241000, China; ahsuqin@263.net (Q.S.); interwuxiao@163.com (X.W.); 2 School of History Culture and Tourism, Jiangsu Normal University, Xuzhou 221116, China; yym412@126.com; 3 Tourism Department, Zhejiang International Studies University, Hangzhou 310023, China; gq7@students.waikato.ac.nz; 4 Business School, Shanghai University of Finance and Economics, Shanghai 200433, China; xiewujie_419@163.com

**Keywords:** local–migrant interaction, ancient tourism town, locals, migrants, grounded theory

## Abstract

Daily interaction is a primary means of understanding social change which is of vital importance for community well-being. In tourism host communities, daily interactions among different resident groups reflect tourism’s effects which are directly related to tourists’ experiences and community harmony. In this exploratory article, grounded theory was applied to analyze daily interactions between locals and migrants in Zhouzhuang, the first ancient tourism town in China, based on in-depth interviews. A model framework was also constructed. The results pinpoint subjective interaction intention and objective interaction opportunities as two influencing factors which operate reciprocally and directly influence locals’ and migrants’ daily interactions. The findings suggest that many locals and migrants experience clear communication boundaries in daily interactions. Both groups’ subjective interaction intentions were influenced by their cultural backgrounds, group perceptions, and original social networks, all of which are difficult to modify within a short time. Therefore, increasing objective communication opportunities is a key way to promote intergroup interaction and integration. These findings provide theoretical and practical implications for tourism host communities’ well-being and sustainable development.

## 1. Introduction

As one of the largest interpersonal communication activities in modern society, the significance of tourism lies first and foremost in cultural exchange and cross-cultural communication [1]. From a sociological point of view, tourism refers to the reproduction of social relations and the spatial extension of social networks [2]. Tourism development has often brought “outsiders”, including tourists, tourism investors, and other tourism practitioners, into many tourism host communities. These newcomers may disrupt a host community’s daily life and interpersonal relationships which are vitally important to retaining traditional culture and the community well-being [3]. Since the 1980s, several scholars have considered the impact of tourism development on residents’ relationships in host communities from the perspectives of anthropology and sociology. However, few studies have closely examined this topic; most focused on either locals [4,5] or migrants (e.g., locals’ sense of deprivation or marginalization and migrants’ sense of isolation) [6]. In less developed areas, capital brought in by investors and an influx of tourism elites can easily cause local residents to become marginalized in tourism destinations [7,8,9]. However, research on local–migrant interaction in everyday life in host communities, especially in developed areas, is greatly limited.

As unique tourism destinations, the allure of tourism ancient towns lies in their rich cultural connotations contained in place specificity as is reflected through traditional foods, regional languages, crafts, folklore, local visual arts, drama, literary references, historic and prehistoric sites, landscape systems, and flora and fauna [10]. Residents’ everyday life and communications, such as their local language, folk customs, habits, and routines, also exemplify these towns. Residents living in ancient towns, including locals and migrants, have become objects of the tourist gaze; their lifestyle and daily communication influence the atmosphere of ancient towns and constitute a towns’ touristic appeal. Such factors are also essential to the sustainable development of these ancient towns and, thus, require further investigation.

China is a traditionally relational society, in which interpersonal communication and social relations constitute important aspects of people’s daily lives. This study took the ancient town of Zhouzhuang, China, as a case study. It is a typical watertown in Kunshan City in the Yangtze River delta, the most developed area in China. Zhouzhuang, an ancient town, has a long history with a profound culture and many celebrities which can be traced to 1086. Surrounded by water and well-developed waterways, Zhouzhuang has always been a famous commercial town in the south of the Yangtze River since the Yuan Dynasty (1206–1368) [11]. As early as 1986, the tourism industry was initiated in Zhouzhuang, which was the forerunner of Chinese ancient town tourism. Then, Zhouzhuang became one of the first historical and cultural towns in China and a national 5A tourist attraction. A large quantity of tourists, businessmen, and holidaymakers swarmed into the ancient town, and the commercial civilization in Zhouzhuang thrived again. We focused on residents’ daily lives in this community, including interactions between locals and migrants. In this study, we aimed to explore different resident groups’ daily interactions in Zhouzhuang, as they face various external shocks. By identifying potential barriers to intergroup interaction, our findings offer theoretical support for the sustainable development of this host community.

## 2. Literature Review

Tourism development has resulted in an influx of “outsiders” pouring into destination communities, mainly tourists and migrants. The local–tourist relationship has been broadly discussed, whereas interactions between locals and migrants have more recently begun to receive particular attention. Williams and Hall (2000) [12] identified two types of tourism migration: tourism labor migration and consumption-oriented migration. Tourism labor migrants refer to those who move from elsewhere to settle in tourism destinations for a certain period, during which they engage in the tourism industry. These migrants can be divided into entrepreneurial migrants and employment migrants based on profession. Consumption-oriented tourism migrants include those who move to a tourist destination either temporarily or permanently for arts and crafts, travel, spirituality, or health-related reasons [13]. Most of these migrants move from cities to rural destinations with beautiful environments or unique resorts and entertainment facilities.

### 2.1. Relationships between Locals and Migrants

Many researchers exploring tourism’s social and cultural impacts have considered the social relationships between locals and tourism labor migrants in tourism host communities. Most work has revealed that tourism labor migrants and local residents are isolated in their daily lives and may experience tension [6,8,14]. For example, McNaughton [15] examined the status of foreign handicraft traders in Pakaram, India. Tourists apparently considered foreign handicraft traders to be hosts. Yet to local residents, traders were neither hosts nor guests but instead uninvited outsiders. Although the vendors’ annual presence resulted in income for the locals, the locals did not welcome them [6]. Because of this exclusiveness, some tourism labor migrants were even disinterested in the host community in which they resided. But in China, Yang’s [16,17] research demonstrated locals’ different attitudes towards tourism labor migrants. In Huangshan, locals adapted to the immigrants’ arrival, so most of them are friendly to tourism entrepreneurial migrants due to the fact of their contributions to the house rental market [16]. In Jiuhua Mountain, locals have distinct attitudes toward tourism labor migrants—welcome or hate—based on their geographical origins and occupations [17]. However, research pertaining to daily interactions between locals and tourism labor migrants remains limited [18]. Relationships between locals and consumption-oriented tourism migrants have come to constitute a popular topic in recent years.

Studies on relationships between local residents and consumption-oriented tourism migrants in host communities have returned inconsistent conclusions. Per Benson’s [19] research on Lot, a famous tourist destination in southwest France, many lifestyle migrants from Britain appeared eager to assimilate into local society, live as authentically as possible, and establish meaningful relationships with local residents. Most migrants usually befriended their neighbors quickly and some even kept in touch with their French neighbors or migrant friends after returning to Britain. In this town, integration into the local community became a driving force for some migrants, and the language barrier between minority groups did not present an obstacle to intergroup communication and integration. These findings were similar to those in Smallwood’s [20] study on British migrants in Astan, France.

However, other research has shown that tourism consumption migrants tend to construct a new “parallel society” in host destinations, where they become separated from local residents. Essentially, they live in an isolated paradise and adhere to their conventional habits. Most have brought their original relationship networks with them and, thus, tend to engage with relatives, friends, or other migrants in daily life rather than establishing new social networks with local residents [21,22]. For instance, Haug et al. [22] conducted a case study of Norwegians living in Spain and found that migrants were indeed isolated from the locals. Norwegians in Spain frequently lived in groups and formed their own migrant communities. Most of them continued to maintain their Norwegian lifestyle while in Spain: they still spoke Norwegian, tended to their gardens, and shopped in typical tourist shops but seldom communicated with native Spaniards. Tourism consumption migrants always developed their social relations in destination communities based on hobbies and leisure activities. However, most regarded these newly built social relations as temporary and superficial according to the relations in their hometowns [23]. Gustafson [21] claimed that consumption-oriented tourism migrants comprised a unique social group between tourists and locals, as these migrants often did not live according to the local customs, rules, and norms. Instead, they tended to conform to tourists in terms of their behavior, perceptions, and experiences within residential communities. They seem to play the part of customers and the destination is more of a business than a community [24]. Consumption-oriented migrants were therefore consistently alienated from the locals in host communities.

### 2.2. Influencing Factors

Previous studies have indicated that although migrants are living and recreating with locals in the same community, most of them, whether tourism labor migrants or consumption-oriented migrants, are isolated from local residents. Friction and conflict among these groups may even arise in some rural parts of suburban areas [7,25]. Contributing factors to this pattern include social exclusion due to the fact of cultural differences, spatial constraints, unequal benefit distribution between locals and labor migrants, and the disparate needs of locals and consumption-oriented migrants.

Research has shown that migrants’ and locals’ distinct cultural backgrounds and values can erect social boundaries in a community. Locals usually develop a sense of self when encountering migrants, effectively distinguishing “us” from “them”. Some tourism labor migrants may eventually gain “local” status, but they will always be “outsiders” to the locals based on cultural background [6,26,27]. Most consumption-oriented migrants also live differently than the locals due to the fact of their discrepant cultural backgrounds which leads migrants to develop separate neighborhoods and to favor in-group communication [22,25].

Spatial constraints present another obstacle. During tourism development, an influx of tourists and tourism investors can infringe on local residents’ private living space and communal space; part or all of such space may be reclaimed for tourism investment [8]. In certain destinations, such intrusion along with consumption-oriented migration has increased consumer and housing prices; skyrocketing costs have begun to drive out locals either voluntarily or involuntarily [28,29]. Migrants also tend to shift from guests to hosts over time, and the crowding-out effect has compromised locals’ lives and recreation space and eroded tourism destinations’ connotations in some host communities [30]. Locals have thus fallen into a relatively weak position and may be susceptible to deprivation which can evoke resentment towards migrants and elicit social tension within host communities [28]. Besides, the seasonal mobility of most migrants also impedes the development of close social knots between them and locals [23,31], while the welfare gap and unbalanced distribution of tourism-related benefits between locals and tourism labor migrants further limits the daily communication and integration of these groups [32].

Consumption-oriented migrants’ needs differ from those of locals [33], creating another communicative barrier. For example, at the seaside resort Barwon Heads, migrants tend to seek a quiet country life whereas residents are interested in recreation and relaxation. These distinct purposes may evoke conflict [26]. Other potentially problematic factors identified in the literature include rural landscape damage, housing vacancies, and community insecurity [25,30].

Community well-being is shaped by residents’ thinking, actions, communication, and interactions in day-to-day life [34]. Undoubtedly, an influx of newcomers (whether labor migrants or consumption-oriented migrants) can weaken the consistency of residents’ social networks and adversely affect host communities’ well-being in a gradual covert but irreversible process [35]. Scholars have noted that social relationships between these newcomers and locals are directly related to sustainable destination development [36]; however, relatively few studies have systematically discussed daily interactions between these groups in tourism host communities, especially in terms of influencing factors. Related findings could provide valuable insight into the sustainable development of tourism destinations.

## 3. Methodology

### 3.1. Study Area

**Figure 1:**
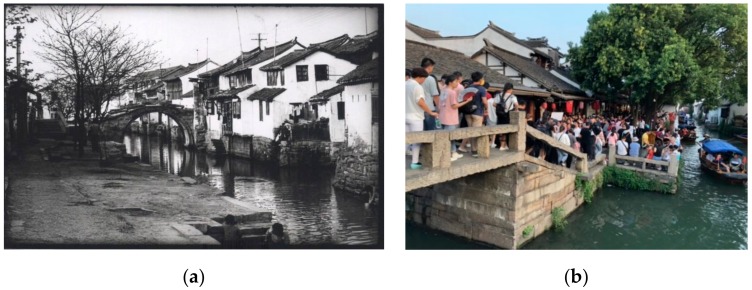
Zhouzhuang ancient town before and after tourism development: (**a**) Zhouzhuang in the 1970s–1980s; (**b**) Zhouzhuang during National Day Golden Week in 2019 (photo (**a**) provided by Kunshan local Chronicles office; photo (**b**) taken by the authors).

Zhouzhuang is an exemplar of ancient tourism towns in China which is adjacent to Shanghai and sits at the center of the Yangtze River delta. Originally, Zhouzhuang served as a living space for local residents and was a typical society based on kinship and geo-relationships. Following tourism development efforts in 1986, Zhouzhuang has been famous for its “stone bridges, creeks, ancient buildings and the village lifestyle”, named “The No. 1 Water Town in China”. In 2018, the town received 5.67 million tourists in total and 15,534 tourists per day within an area of 0.47 km^2^. Many migrants have also been drawn to invest, work, or settle in Zhouzhuang. The ancient tourism town has since become a public space occupied by tourists, migrants, and local residents (see Figure 1).

The residents of Zhouzhuang can now be divided into locals (i.e., registered permanent residents) and migrants (i.e., those who have lived in Zhouzhuang for more than six months and possess a temporary residence permit) according to household status. The town hosted a registered population of 4623 locals and 1943 migrants by the end of November 2017 based on statistical data; however, the proportion of migrants is larger than this estimate. Many locals are registered in the ancient town but live outside of it, while other migrants have not registered at all. Migrants include tourism labor migrants and their families along with some consumption-oriented migrants. Although certain isolated, entrance-guarded communities exist away from the scenic area and are mainly inhabited by consumption-oriented migrants, few of them live in Zhouzhuang full time. They tend to visit Zhouzhuang to enjoy holidays or during weekends and festivals. These visitor trends have led to high vacancies in these communities. As such, the proportion of consumption-oriented migrants in the town is somewhat low. Most tourism labor migrants come from neighboring provinces and cities such as Jiangsu, Anhui, Henan, and Zhejiang. Consumption-oriented migrants often travel from nearby cities including Suzhou and Shanghai and are often attracted by the town’s idyllic and tranquil atmosphere.

**Figure 2:**
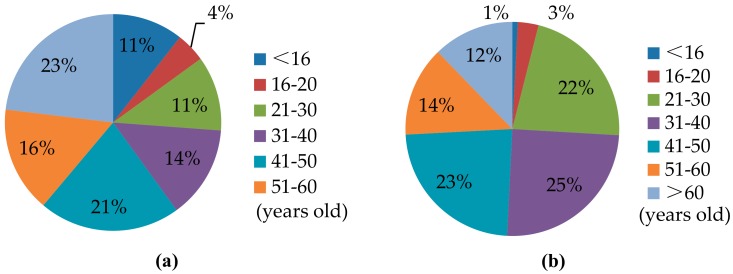
Age distribution of locals and migrants in Zhouzhuang in 2017: (**a**) Locals; (**b**) migrants.

The locals and migrants in Zhouzhuang also differ substantially in terms of age and occupation. Locals tend to be older: 1067 retirees over 60 years old were living in the ancient town at the end of 2017, accounting for 23% of the registered population. Interviews conducted for this study revealed that locals living in the scenic tourism areas tended to be retirees. Comparatively, only 12% of migrants were over 60 years old, and 87% were of working age (i.e., between 16 and 60 years old; see Figure 2). The occupational composition in Zhouzhuang also demonstrated clear differences. Except for elderly retirees and school-age children, most young and middle-aged locals reported working at either Zhouzhuang Tourism Development Corporate Ltd. (ZTDC, a collectively owned enterprise) or public institutions in the town; only a few were running tourism businesses at the time of this study. By contrast, most migrants were running tourism businesses or working as tourism servants near scenic areas.

### 3.2. Data Collection

Data for this study consisted of secondary data and data gathered through fieldwork. Secondary data included published papers and archival data such as resident demographics, migrant registration, government documents, and reports. The second author’s research team began monitoring tourism development in Zhouzhuang in 2002, resulting in rich archival data related to the town’s expansion, spatial evolution, and population constitution.

Fieldwork in Zhouzhuang was conducted through face-to-face interviews, informal conversations, and non-participant observations between 2016 and 2017. First, the research group visited several governmental agencies where some officers were interviewed to collect recent statistics on residents’ constitution in Zhouzhuang along with information about related government policies and documents. Next, 54 residents, including locals and migrants, participated in in-depth interviews in November 2017 regarding their everyday lives, intergroup communication, and perceptions of their daily relationships (Table 1). With the help of the director of neighborhood committee, all the interviews were conducted at the interviewees’ homes or in their shops. First, the respondents were asked to talk about their family, work, and social relations before they engaged in tourism industry (locals) or migration (migrants); then, they were required to describe their daily life routines, main activities, and daily interactions in groups and among groups at present; how they perceived life and daily interactions; how they saw each other; and the reasons for engaging in interactions. Finally, they were asked, when they encountered difficulties, whom would they turn to? Why? The interview times ranged from 1 to 2 h per person. Collected materials included interview notes, audio recordings, and photos. The authors also visited residents’ homes, vegetable markets, and community activity centers to obtain more details about residents’ lives and daily interactions, mainly through non-participatory observations and informal conversations. In addition, the first author (Zhang) was actively involved in the editorial team for a book entitled “Imaging Chronicles of Zhouzhuang Ancient Town” and participated in interviews with tourism stakeholders from 2017 to 2019. Detailed oral material on the development history and residents’ participation in Zhouzhuang tourism were thus obtained. The authors also kept in touch with key persons throughout the data collection process via WeChat instant messaging to obtain supplementary data and verify information as needed.

### 3.3. Research Method

Residents’ interactions are closely related to, and an important part of, their daily lives. Such interactions are shaped by an array of subjective and objective factors which are highly complex and difficult to quantify or measure. Therefore, an interpretive, naturalistic qualitative approach was deemed most appropriate for this study, given its focus on observing people in natural settings to understand individuals’ social behavior [37,38].

Creswell described five popular qualitative approaches: narrative research, phenomenology, grounded theory, ethnography, and case study [39]. Among these, grounded theory was chosen to explore daily interactions between locals and migrants in the host community of Zhouzhuang; this approach is useful for analyzing people’s actions, interactions, and social processes based on data collected from individuals.

Grounded theory has been defined as a theoretical rendering of reality [40]. It is an ideal qualitative research method for developing theories rooted in empirical data. As a revolutionary part of the qualitative tradition, the hallmark of grounded theory is the comprehensiveness of its procedures, techniques, and assumptions related to the discovery of practical theory [41]. Coding is the cornerstone of this theory, referring to a process of abstracting conceptual categories from empirical data and moving towards theoretical generalizations [38]. In grounded theory, coding consists of three phases: open coding, axial coding, and selective coding. Open coding involves analyzing all original material item by item, including field notes, photos, textual data, and reports, after which preliminary categories are extracted and labeled; in other words, themes are identified from raw data. Axial coding occurs next, in which researchers seek to find and establish connections among categories or themes. This step involves clustering primary themes to determine causes and consequences along with conditions and interactions. During selective coding, all major themes are arranged into a storyline to develop a theoretical explanation of the research problem. Nvivo12.0 was applied for text analysis in this study. The software was also used to code all materials step by step in an attempt to construct a framework of daily local–migrant interactions in Zhouzhuang to illustrate the status of their daily communication and any direct influences.

Data triangulation was used to improve the credibility and dependability of our findings [42]. The perspectives residents shared in interviews were cross-referenced to notes taken during the researchers’ observations, secondhand documents, and survey data from the other two team members’ research on the floating process of migrants and the social distance among Zhouzhuang residents [43,44]. During interviews, the researchers paid particular attention to different stakeholders’ viewpoints (e.g., residents and committee staff, tourism and non-tourism practitioners, ZTDC staff, and other residents) to ensure data reliability. Then, the data were further compared and verified based on various materials including oral materials, government documents, and related articles.

## 4. Coding Results

The researchers conducted 54 interviews, including 19 with local residents and 35 with migrants. All materials were numbered from 1 to 54, in which L1, L2, … L19 denoted locals’ materials, and M1, M2, … M35 denoted migrants’ materials (Table 1). Numbering was followed by open coding, axial coding, selective coding, and finally construction of the theoretical model. Then, we performed a theoretical saturation test; sample saturation is a key step in grounded theory. In this case, interview data from five participants (i.e., L3, L14, M5, M18, and M35) were randomly drawn to verify core-concept saturation.

### 4.1. Open Coding

Open coding is an operational process that deals with relevant materials in fragments and extracts themes from them. To minimize personal bias and stereotypes in this process, the authors were split into two groups to compile interview materials sentence by sentence. Next, both sets of coding results were compared, and any inconsistencies were reviewed until a consensus was reached. Thirty-five concepts regarding daily local–migrant interactions in Zhouzhuang were initially abstracted, which were then integrated into 15 categories by referring to relevant literature (Table 2).

### 4.2. Axial Coding

Based on the results of open coding, axial coding was used to establish relationships among codes and analyze relevant categories. The 15 identified categories were collapsed into 6 as follows: cultural background (AC1), group perceptions (AC2), original social network (AC3), subjective interaction intention (AC4), objective interaction opportunities (AC5), and interaction states (AC6) (Table 3). We referred to relevant research to improve the theoretical basis and generality of these categories.

### 4.3. Selective Coding

**Figure 3:**
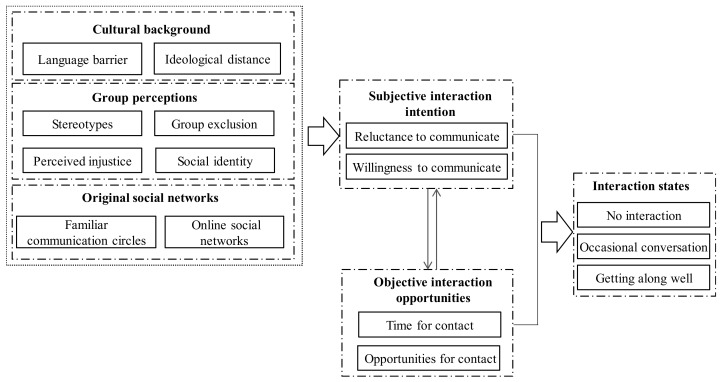
Model of daily local–migrant interaction in Zhouzhuang.

Selective coding involves exploring intrinsic relationships among attributes and deriving storylines to develop theoretical explanations for problems. Through in-depth analysis, logical relationships were identified among the six main categories. “Influencing factors in the two groups’ daily interactions” was the core category. Residents’ daily interactions appeared to be shaped by subjective interaction intention and objective interaction opportunities; therefore, “subjective interaction intention” and “objective interaction opportunities” were identified as influencing factors which can also influence each other and lead to three interaction states: no interaction, occasional conversation, and getting along well. The two groups’ subjective interaction intentions were determined by their cultural backgrounds, group perceptions, and original social networks. Based on these relationships, an analysis model of daily local–migrant interaction in Zhouzhuang is illustrated in Figure 3.

### 4.4. Theoretical Saturation

Theoretical saturation indicates that reasonable correlations have been established among all categories, and new concepts have ceased to emerge. We performed coding analysis on the five reserved pieces of interview material (see Section 4) and identified relevant categories to assess theoretical saturation. Of the five pieces, two were from local residents (L3, L14) and three were from migrants (M5, M18, M35).

For L3, we identified two categories, namely, “no interaction” and “opportunities for contact”. Sample statement: “We never interact with ‘outsiders’ … They are always busy with their business. We usually go to buy vegetables in the morning, then stay home all day. We don’t go outside during the day, only to Nanhu Park or Jiuqu Bridge to exercise in the morning and evening...”

For L14, the categories of “group exclusion” and “stereotypes” were identified. Sample statement: “The arrival of migrants must influence local residents’ business. The competition among businesses in Zhouzhuang is fierce now. [Migrants’] arrival makes it harder to make a profit. Locals benefit from tourism much less. There are more and more migrants in Zhouzhuang, most of whom are complicated and have poor character…”

For M5, two categories were identified: “stereotypes” and “original social networks”. Sample statement: “…Locals are bad, and Southerners are not human … Normally we have little time to rest; we only go back [to our] hometown to meet our family and friends when we have free time.”

For M18, the “language barrier” and “no interaction” categories were identified. Sample statement: “I’m not used to hearing the local dialect here, and I don’t understand it either. I have few chances to talk with locals; they don’t seem to talk with us either…”

For M35, “cultural background”, “original social networks”, and “group exclusion” categories were identified. Sample statement: “We are all fellow villagers from Anhui province, so we always get along well … I have been in Zhouzhuang for several years. In my opinion, locals here are a little exclusive. They always feel [like it’s] unfair when we come to do business here...”

As indicated, no new categories emerged from the preceding text analysis. At the end of this process, we reviewed other team members’ interview data on this topic to evaluate concept-based saturation. We found that 46 residents mentioned daily interactions, and no new categories were identified. The six core categories thus appeared to have reached theoretical saturation.

## 5. Results

Based on grounded theory, we constructed a conceptual model of daily local–migrant interactions in Zhouzhuang (Figure 3). This model mainly consists of influencing factors and interaction states between the two groups.

### 5.1. Influencing Factors

#### 5.1.1. Subjective Interaction Intention

Subjective interaction intention refers to one group’s willingness to communicate with another group, derived from the psychological power source that promotes interpersonal communication. According to interview findings, most locals and migrants exhibited relatively high interaction intentions in Zhouzhuang; this finding is consistent with Huang’s results regarding social distance between locals and migrants in Zhouzhuang [44]. Slightly more than half (57.89%) of locals and 60% of migrants were willing to interact with each other, whereas others were reluctant or found such interaction unnecessary. A migrant from Taiwan said:

“I am from Taiwan, I was sent here by the head office. We have six Taiwanese here. We work and live together. We always go back home on holidays. It seems that we don’t have to communicate with [locals]…”

(M13)

Interaction intention was shaped by individuals’ cultural backgrounds, group perceptions, and original social networks.

##### Cultural Background

Similar cultural backgrounds can lead to common community foci, promoting intergroup communication and integration; comparatively, different cultural backgrounds may hinder intergroup communication [31]. This distinction held true in Zhouzhuang. Dissimilar cultural backgrounds represented an important factor that compromised intergroup communication in the ancient town, namely, reflected in linguistic differences.

Social interaction encompasses communication activities between subjects and objects, which rely on language and other symbols. Language is one bridge in group interaction [45] and is pivotal to how migrants see themselves and how others see them [46]. Zhouzhuang’s dialect falls under the Wu dialect within the Taihu cluster which differs substantially from Mandarin. Most migrants in Zhouzhuang can neither speak the dialect nor understand it. One remarked, “Speaking of chatting with locals, I don’t like it. I can’t speak the Zhouzhuang dialect. Although I am also from Jiangsu Province (Zhouzhuang is in the same province), the dialect here is quite different. It sounds awkward, never mind using it…” (M32). A local noted, “I am not willing to chat with ‘outsiders’ because I can’t speak Mandarin, [and] they don’t understand me either. Communicating with them is so inconvenient…” (L15). The local dialect clearly poses a major barrier to local–migrant communication in this town.

Ideological distance further reflected migrants’ and locals’ distinct cultural backgrounds. Zhouzhuang is the home of “Wu culture”. Before the town’s tourism development, it was isolated from the outside world due to the poor transportation. The local culture and characteristics thus became relatively prominent, constituting the ancient town’s primary appeal. The ideological distance between locals and most migrants in Zhouzhuang thus remains prevalent. During interviews, some migrants mentioned that they did not understand the culture in Zhouzhuang, especially local living habits and, therefore, chose not to communicate with locals. This reluctance presents another communicative barrier.

Given the area’s extensive tourism development, locals in Zhouzhuang are becoming increasingly tolerant of foreign cultures and migrants. A local said, “I would like to interact with ‘outsiders’... Tourism is developing very well now in Zhouzhuang, and there are more and more ‘outsiders’ working and living here. Hence, if we get along well, Zhouzhuang will become better and better” (L19). Some migrants who had worked and lived in the ancient town for a relatively long time, especially those who planned to settle there, appeared quite willing to interact with locals despite social and cultural distance.

##### Group Perceptions

Social perceptions refer to people’s perceptions of individual or group characteristics. In this study, we use the concept of group perceptions to characterize locals’ or migrants’ perceptions of each other, including stereotypes, group exclusion, perceived injustice, and group identity.

Stereotypes are mental constructions, specifically group members’ oversimplified impressions of a particular type of person or thing given a lack of familiarity [47]. According to interviews, some locals held the stereotype that “migrants are unreasonable… (L15)”; “They are good at making money … They only believe in money [and] don’t care about the environment of the ancient town…” (L12). Migrants often remarked, “Locals are rich… They have a comfortable life without hard work…” (M27). These and other stereotypes can impede interaction between locals and migrants in daily life.

As more migrants flow into Zhouzhuang, some locals have developed a strong sense of deprivation. Some residents lamented, “[Migrants] have their ways, and they are good at earning money. All the money was earned by them…” (L12). Certain residents also expressed envy towards these ”outsiders” who ran successful businesses in the ancient town: “…They are all ‘outsiders’, [and] we feel helpless…” (L14). This sense of deprivation led some residents to reject migrants which many migrants readily perceived. One owner of a guest-house, who came to Zhouzhuang in 2001, said, “I have been here for many years. I feel that most locals have a sense of xenophobia. They always feel uncomfortable when migrants come and run business in the ancient town…” (M35). Similarly, a female migrant private entrepreneur explained, “[Locals] still discriminate against us…” (M16).

Locals’ sense of deprivation and migrants’ sense of discrimination led to group exclusion. Many migrants, especially businessmen, expressed a strong sense of injustice:

“There must be a difference between us and the locals when running businesses here…we aren’t treated like locals here by executives from government or ZTDC (A collectively owned enterprise responsible for the operation and management of Zhouzhuang’s tourism industry, in which most staff are locals]…”

(M25)

This sense of injustice strengthened migrants’ perceived exclusion and greatly diminished their willingness to communicate with locals. However, locals and migrants began to identify with each other after reaching an understanding; group identity could thus promote intergroup communication. For example, a local clerk in the community said, “I find [migrants] are nice after meeting them…” (L9). A migrant noted, “At first I was not used to the lifestyle here, but now I gradually feel comfortable…. Neighbors are nice too; I like to communicate with them…” (M17).

##### Original Social Networks

Original social networks are mainly based on relative and blood relations. Research has shown that such networks are important to migrants’ migration and their life in a given destination [48,49]. This is particularly true in traditional Chinese society, where the foundation of daily interaction is spontaneous communication based on kinship and natural emotion [50]. These characteristics tend to manifest as kinship and a regional network, including residents’ familiar communication circles and online social networks in the case of Zhouzhuang.

Most locals and migrants in the ancient town have familiar communication circles based on similar cultural backgrounds of blood and kinship; these relationships constitute their daily communication circles, which further reduce their willingness to interact. A migrant female waitress said

“More than 10 families in our village are doing business here. All of my brothers and sisters are here, too. We work together every day. I seldom feel alone…If I need help, I will turn to them…”

(M15)

Locals in Zhouzhuang enjoy drinking tea. In the afternoon, many retired older people visit recreation centers for the elderly to drink tea, exercise, and meet friends. A retired local man said, “I like having a cup of tea and chatting with old neighbors and colleagues. We are familiar…” (L7)

Online social networks have become an indispensable part of daily life [51] which cannot be neglected in Zhouzhuang. Locals’ online networks with former neighbors who have moved away and networks between migrants and their relatives and friends in their hometowns may create a communication gap and reduce the need for intergroup communication. A newly arrived businessman in town remarked, “I have no friends here. I chat with my relatives and friends online every day…” (M1).

Similar lifestyles and habits tend to promote cohesive bonding [52]. Locals and migrants can more easily develop and expand their everyday networks based on similar languages, shared customs, and living habits. However, these networks hinder intergroup communication to some extent.

#### 5.1.2. Objective Interaction Opportunities

There are two basic conditions for group interaction: consistent interactive symbols and social contact. Interactive symbols can be verbal or non-verbal, and social contact can be direct or indirect (e.g., via tools) [53]. Linguistic differences may limit communication between locals and migrants in Zhouzhuang as discussed above. Interviews revealed two factors that further influenced local–migrant interaction, namely time and chances for contact.

Regarding time for contact, many tourism practitioners (whether locals or migrants) noted they had little leisure time given the year-round and 24 h nature of tourism reception. During interviews, a typical practitioner response was, “I am very busy with tourism business; I have no time to chat…” (M4). Migrants’ lifestyle of “coming and going” on a regular basis also contributed to a lack of contact among the two groups [31]. A local said, “The stores here change frequently. We have no time to get to know [anyone]. Every time I see a newcomer, I just say hello…” (L11). Notably, however, some residents with fixed working times consistently interacted after work through either in-group or intergroup communication, such as playing cards or having dinner together.

**Figure 4:**
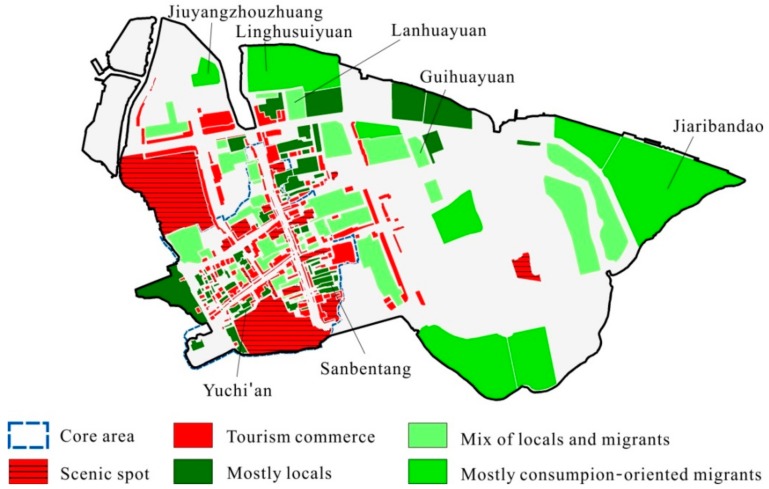
Distribution of residential space in Zhouzhuang.

In terms of opportunities for contact, some locals and migrants live together in the ancient town, but more than half live in concentrated communities. As such, there are few chances for contact between many migrant tourism practitioners and locals, especially retirees, due to the separation in residential space and daily activity space. Figure 4 depicts the general distribution of residential space in Zhouzhuang. The core area consists primarily of tourism space with some public houses situated off of commercial streets. These public houses are mainly occupied by old locals (e.g., Sanbentang or Yuchian) who tend to stay inside during the day and have no contact with migrants. Some traditional unit communities are situated outside scenic areas (e.g., Guihuayuan and Lanhuayuan) with live-in public institution staff or newly built commercial residential buildings occupied by a mix of locals and migrants. During the day, most of these labor migrants are busy with their businesses in the core area of town. An “outside” local explained, “As soon as [migrants] arrived, they set up businesses in the scenic area. We have no contact with ‘inside’ [migrants]…” (L6). Few consumption-oriented migrants live in Zhouzhuang full time; they mainly live in gated communities outside the core scenic areas such as Jiuyangzhouzhuang, Linghusuiyuan, and Jiaribandao. Most of these migrants are therefore isolated from locals and tourism labor migrants.

Participating in tourism, tourism cooperation, and renting houses could increase opportunities for intergroup contact which could then promote group interaction and integration. For example, a local retired travel agent remarked, “…Before participating in the tourism industry, I mainly communicated with local people. But now I have become acquainted with a lot of migrants…” (L18). An owner of a tourism shop said, “I rent a house from local people, and I live with them. Of course, we see each other every day…” (M11).

We also found that shared religious beliefs and participation in religious activities provided opportunities for local–migrant contact in Zhouzhuang. A migrant businessman said, “I believe in Christianity, and I participate in church activities regularly. I get to know a lot of people, locals, and migrants…” (M19).

#### 5.1.3. Interplay of Subjective Interaction Intention and Objective Interaction Opportunities

Subjective interaction intention constitutes the direct dynamics of intergroup interaction, whereas objective interaction opportunities comprise a basic condition of interaction. Interviews revealed the interplay among these interaction dimensions between locals and migrants in Zhouzhuang. On one hand, locals or migrants with low interaction intentions tended to avoid encroaching upon each other’s activity space, limited contact, and communicated daily with members of their own group. This pattern echoes Quinn’s [54] findings in the historic city-center of Venice. On the other hand, locals and migrants who came into frequent contact (e.g., by participating in the tourism industry, religious activities, or renting a house) were more tolerant of and kind to each other. They also tended to understand one another better, which enhanced their willingness to communicate. Some locals and migrants mentioned that, “I find [that members of the other group] are very nice after meeting them...” (L9). Group prejudice therefore diminished gradually, with some individuals even surmounting the language barrier to facilitate intergroup interaction.

### 5.2. Interaction States

**Figure 5:**
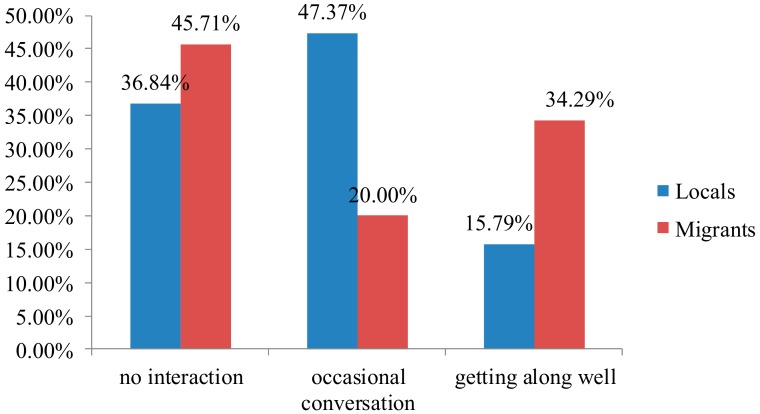
Interaction states between locals and migrants.

Daily local–migrant interaction in Zhouzhuang consisted of three states amidst subjective communication intentions and objective communication opportunities: no interaction, occasional conversation, and getting along well (Figure 5).

In many cases, the two groups were separated within daily life. Most locals (84.21%) and 65.71% of migrants indicated they had little interaction in their day-to-day workings. Some reported, “Sometimes we chat with each other, but [there is] no friendship, just ordinary chitchat...” (L16). Additionally, 36.84% of locals and 45.71% of migrants reported having no contact with each other in everyday life. An old villager explained, “I always chat with old neighbors. I don’t speak to migrants…” (L1). A migrant shop owner pointed out, “I have no relatives or friends here … and I seldom talk with locals...” (M8). However, another 15.79% of locals and 34.29% of migrants thought they got along well. For example, an elderly fisherwoman said, “[Migrants’] quality is high, and some of them are also easy to get along with. Our relationship is quite harmonious…” (L19). A migrant waitress from Anhui province remarked, “I get along well with locals. We always play cards or mahjong together…” (M15).

Through interviews, we found that elderly retirees, who comprised 23% of locals, and the staff of enterprises or public institutions generally maintained daily contact with old friends, relatives, neighbors, or colleagues. Most young and middle-aged locals who participated in tourism had established a daily communication network related to work which included locals and migrants. Migrants tended to maintain close contact with their relatives and fellow townsmen, often striving to establish business networks. No overt conflict or antagonism emerged among the two groups; however, intergroup communication seemed relatively limited and mostly confined to business contacts with little neighborly chitchat. Most locals and migrants, especially retired locals and consumption-oriented migrants, appeared deeply segregated. Even so, some locals and migrants reportedly got along well, especially those with many opportunities for contact (e.g., living together, sharing a religion and participating in religious activities regularly, or sharing business contacts and cooperation).

## 6. Discussion

Interaction underpins group integration. The results of our study revealed that three states characterized daily local–migrant interaction in Zhouzhuang: no interaction, occasional conversation, and getting along well. Many locals and migrants had limited interaction in everyday life, indicating clear communication boundaries and isolation. Interaction between locals and migrants of different ages and occupations was also distinct. For instance, most elderly individuals’ intergroup communication was quite limited, whether among locals or migrants. Consumer-oriented migrants represented another relatively isolated group. Yet among young and middle-aged individuals, intergroup communication was influenced by their tourism industry engagement. This finding is similar to that identified in prior work [21,22,25,55].

Subjective interaction intention and objective interaction opportunities were primary direct influences in daily interactions between locals and migrants in the ancient town. Subjective interaction intention was shaped by individuals’ cultural backgrounds, group perceptions, and original social networks which also emerged in previous studies on intergroup communication in tourism communities [6,25].

Differences in cultural backgrounds have been consistently identified as major obstacles to intergroup communication and integration, and tourism host communities are no exception [6,25,31]. Cultural differences, such as those involving language and ideological conceptions, also limited daily interactions between locals and migrants in Zhouzhuang. However, based on similar cultural backgrounds of blood relations and kinship, both groups in the ancient town maintained their original social networks. These connections formed their daily communication circles and decreased intentions to communicate, similar to Carson’s [31] study in Sweden.

The dimension of group perceptions is quite complicated in Zhouzhuang, consisting of group stereotypes, group exclusion, perceived injustice, and social identity. The first three factors can expand the social distance between groups and diminish efforts in intergroup communication. However, as tourism development has continued, some locals and migrants have begun to identify each other. Another noteworthy finding concerns the lack of objective interaction opportunities due to the limited communication time and contact chances as a result of daily isolation between locals and migrants in Zhouzhuang. Elderly locals, tourism labor migrants, and consumption-oriented migrants are particularly separated and have little contact in daily life. However, those who see each other frequently when participating in tourism, business, religious activities, or shared home space may begin to interact more often. Some locals and migrants even reported having established a good relationship that shifted from work-oriented interactions to daily interactions. Furthermore, there have been some cases of intermarriage between locals and migrants in Zhouzhuang, and some migrants have bought houses and settled there. These consequences have arisen from close intergroup interaction and a high level of group identification. Such trends also reflect the interaction between the two groups’ subjective interaction intentions and objective interaction opportunities.

Whereas migrants are considered “outsiders” in host communities, tourists consider them community hosts along with the locals. Tourists interact with migrants, and they become objects of the tourist gaze. As such, daily interactions between locals and migrants are directly related to tourists’ destination experiences and play important roles in the sustainable development of such communities. Our results demonstrated that many locals and migrants in Zhouzhuang appeared isolated from each other in daily life due to the low interaction intention and few interaction opportunities. However, different from the marginalization of locals in some tourist destinations [5,56,57], locals and migrants in Zhouzhuang shared relatively equal status and occupied different branches and departments of the tourism economy despite exhibiting certain forms of residential and communication-based isolation in daily life. Essentially, they lived together peacefully but tended to keep their distance from one another; most of them favored their original social networks of friends and relatives or formed various small groups. These patterns are quite different from those identified by McNaughton [6] in India. Compared with the fierce local–migrant conflict there, the relationships between locals and migrants in Zhouzhuang often appeared more polite. Interviews with locals and migrants suggested that most were satisfied with their life and daily interactions in Zhouzhuang. Although the focus of town life and interactions had changed with tourism expansion, these changes seemed normal against the backdrop of the development of the social economy and tourism commercialization. Locals and migrants gradually adapted to this process. However, the question of how best to promote intergroup communication and community integration is still worth pondering.

Our study suggests that although subjective interaction intention and objective interaction opportunities are core influencing factors in the interactions between locals and migrants in Zhouzhuang, interaction intention is shaped by residents’ cultural background, group perceptions, and original social networks; these factors are determined by individuals’ social status, which is a form of objective existence. These characteristics also apply to nearly all intergroup interactions and are therefore difficult to modify within a short time. Yet individuals who had established good intergroup relationships tended to engage in frequent contact in daily life. Therefore, increasing objective communication opportunities between locals and migrants in Zhouzhuang, including time and opportunities for contacts (and particularly chances for benign interaction), could directly abolish group stereotypes and prejudices to encourage intergroup interaction and integration. This supposition is consistent with Allport’s [58] “contact hypothesis” which posits that a lack of contact and communication among groups leads to insufficient information and poor mutual understanding; these barriers constitute major drivers behind group prejudice, stereotypes, and other intergroup conflict. Conversely, frequent intergroup contact can promote information exchange and understanding between groups, thus encouraging better intergroup relations.

## 7. Conclusions

This study analyzed daily interactions in a destination community from locals’ and migrants’ perspectives. We extracted several main categories of interaction states and influencing factors via qualitative analysis and constructed a framework of daily intergroup interaction. Our findings enrich the body of literature on social relationships in tourism host communities and community well-being. In this study, we paid particular attention to internalization factors with respect to intergroup interaction and contributed to a systematic understanding of such interaction in tourism host communities.

We attempted to assess daily intergroup communication in a heritage tourism destination within a developed region. Traditional ancient towns tend to be commercially integrated locations that are home to a relatively large migrant population. After tourism development, such towns become tourism destinations but remain residents’ home. Tourism ancient towns are therefore distinct from other types of destinations; the interaction and integration of different groups directly influence community well-being as well as sustainable tourism development. We chose Zhouzhuang, the first town to develop a tourism industry in China, as a case study to examine communication relationships in everyday life which hold great importance for the long-term well-being of other heritage tourism communities. Meanwhile, it is also meaningful for the improvement of residents’ quality of life in tourism host communities from a practical point of view.

In terms of managerial implications, we can recommend several policy revelations. Per the contact hypothesis, there are four prerequisite conditions for optimal intergroup contact: equal group status; common goals; intergroup cooperation; and support from authorities, laws, or customs [51]. We believe that promoting peaceful cooperation between locals and migrants at the government level, including formulating community goals and norms that are approved by both groups, presents an optimal path to strong intergroup relations. These actions could also encourage benign development of destination communities. First, communities’ neighborhood committees and other government agencies can create opportunities for locals and migrants to interact through community activities; doing so would dismantle the communication barrier between them, promote gradual small-group integration, and achieve assimilation and sustainable community development. Second, reciprocity is the basis of strong social relationships [59]. Related departments could therefore act as matchmakers to promote multi-form collaboration and mutual assistance among community merchants, thereby encouraging the development of social networks based on reciprocal cooperation to promote sustainable community development. In addition, the interaction and integration of different community groups require relevant policies and system-wide protections. For example, residential space planning typically follows the principle of “mixed inhabitancy” to reduce interaction isolation caused by residential separation. Community participation could be promoted in various ways, potentially decreasing locals’ intentions to leave due to the fact of tourism’s excessive dependence on external investment and migrants. Huttasin [60] strongly recommended whole-community participation in the tourism industry to encourage sustainable development. In indigenous areas like ancient towns, it can lead to natural and cultural conserving while improving local living standards [61]. Recently, Zhouzhuang’s local government and ZTDC have advocated for residents to develop home tourism, representing a genuine effort to boost local residents’ participation in the tourism industry.

This study has certain limitations. Residents’ daily communication in tourism host communities is influenced by destinations’ cultural and historical backgrounds, modes of tourism development, and many other factors. Given our research conditions, we focused on everyday interactions on the basis of interview data and secondary statistical data regarding a single case. Studies of other towns may elicit different results. Scholars may wish to employ a combination of qualitative and quantitative methods in future research to compare multiple cases using questionnaires, interviews, and oral history; this approach would engender a more comprehensive understanding of residents’ daily communication and intergroup integration in tourism host communities. Moreover, individuals’ decisions about whether to engage in tourism can greatly influence intergroup interaction in tourism communities. Further research could address intergroup interaction by type. Today’s ongoing social, economic, and cultural evolution—coupled with more intensive population mobility, information flow, and virtual communication—also affect residents’ daily communication. Therefore, future studies should consider community residents’ daily interactions from a broader perspective.

## Figures and Tables

**Table 1:** Demographic characteristics of interviewees.

Serial Number	Gender	Age	Occupation	Serial Number	Gender	Age	Serial Number
L1	Female	~60	Retired	M9	Female	36	Guest-house assistant
L2	Male	92	Retired	M10	Female	19	Shop assistant
L3	Female	≥60	Retired	M11	Female	38	Shop owner
L4	Male	31	Innkeeper	M12	Male	28	Company employee
L5	Male	77	Retired	M13	Male	~30	Skilled worker
L6	Male	≥60	Innkeeper	M14	Female	~40	Clothes shop owner
L7	Female	≥50	Retired	M15	Female	~40	Waitress
L8	Male	≥20	Government staff	M16	Female	34	Shop owner
L9	Male	≥30	Government staff	M17	Male	60	Craftsman
L10	Female	50	Farmer	M18	Female	29	Calligraphy Shop owner
L11	Male	41	Shop owner	M19	Female	29	Shop owner
L12	Male	≥60	Innkeeper	M20	Male	39	Restaurant owner
L13	Female	≥30	Barkeeper	M21	Female	37	Clothes shop owner
L14	Female	47	Freelancer	M22	Female	36	Shop operator
L15	Male	≥65	Retired	M23	Male	30	Tea shop owner
L16	Male	≥70	Retired	M24	Female	36	Shop assistant
L17	Female	~45	Community staff	M25	Female	≥20	Tea shop operator
L18	Female	≥50	Guide in ZTDC	M26	Male	52	Guest-house owner
L19	Female	≥50	Fisherman	M27	Female	30	Shop owner
M1	Female	≥20	Guest-house operator	M28	Male	64	Retired
M2	Male	≥40	Restaurant owner	M29	Female	≥45	Waitress
M3	Female	≥60	Waitress	M30	Male	unknown	Actor
M4	Male	≥30	Restaurant owner	M31	Female	unknown	Fruit shop owner
M5	Male	≥20	Shop assistant	M32	Female	41	Shop owner
M6	Female	~45	Innkeeper	M33	Female	30	Clothes shop owner
M7	Female	~30	Shop assistant	M34	Male	31	Shop owner
M8	Male	≥30	Shop owner	M35	Male	≥40	Guest-house owner

ZTDC: Zhouzhuang Tourism Development Corporate Ltd.

**Table 2:** Examples of open coding analysis.

Categorization	Conceptualization	Excerpt of Interview Material
A. Language barrier	a1. Migrants can’t understand and speak dialect	I can’t speak the dialect…
		They don’t understand what I am saying, and I can’t speak Mandarin…
	a2. Locals can’t speak Mandarin	
B. Ideological distance	b1. Variation between ideas	Because of differences in ideology…
	b2. Don’t understand each other	
		…we don’t know anything about them.
C. Stereotypes	c1. Locals are terrible and impersonal	… they [locals] are terrible…
		Southerners [locals] are very impersonal…
		I think some migrants are unreasonable.
	c2. Migrants are unreasonable	
D. Group exclusion	d1. Migrants feel discriminated against	Most locals look down upon us [migrants]…
		Most tourism businesses are run by migrants, and money is earned by them, too…
	d2. Locals’ sense of deprivation	
E. Perceived injustice	e1. Migrants’ perceptions of unfair management	Locals can open shops first, and then apply for a business license, but [migrants] can’t.
		Those villas are expensive for [locals], but they are a drop in the bucket for Shanghainese [migrants].
	e2. Perceptions of the wealth gap	
		…most locals have good socioeconomic standing.
F. Social identity	f1. Quite good	Some migrants are quite good, and they are relatively easy to get along with…
	f2. Friendly	
	f3. Locals and migrants are the same	
		We are the same, locals here are friendly…
G. Familiar communication circles	g1. Old neighbors	I usually chat with my old neighbors. We always gather to have amiable conversations.
	g2. Fellow townsmen parties	
		We have many fellow townsmen here. We get along well, and we host parties together.
H. Online social networks	h1. Contact through instant messenger	I always contact my friends in my hometown through WeChat or QQ. I don’t feel lonely.
	h2. Communicate online	I usually communicate online with colleagues at other inns.
I. Reluctance to communicate	i1. Do not turn to the locals for help	If I’m having any difficulties or problems, I hardly think of [the local people]…
	i2. Do not need to interact	
J. Willingness to communicate	j1. Being willing to communicate	I like dealing with migrants.
	j2. Make friends	
		I am willing to make friends with locals.
K. Time for contact	k1. Do not have time to communicate	I run two shops. I don’t have time to chat……
	k2. Chatting after work	We always chitchat together when we’re not working.
L. Opportunities for contact	l1. Separate spaces for living and activity	[Migrants] are busy, always go back home late at night, and we always go to sleep at that time.
	l2. Participating in church activities together	
		We live together, so we talk every day…
	l3. Business cooperation	
	l4. Living together	The tourism shops change frequently…
	l5. Rapid flow of migrants	
M. No interaction	m1. Do not get in touch with each other	We don’t get in touch with migrant businessmen…
	m2. Have no contact	…We don’t contact the locals…
N. Occasional conversation	n1. Only chat together	Sometimes we talk together, but we rarely have time for much fraternizing.
	n2. Daily greeting	
		I just say hello to familiar migrants…
O. Getting along well	o1. Good relationship	We get along well with each other…We also share meals sometimes.
	o2. Interact every day	
	o3. Help each other	
		I communicate with [locals] every day.

**Table 3:** Main and subcategories in axial coding analysis.

Main Categories	Categories
MC1 Cultural background	A. Language barrier
	B. Ideological distance
MC2 Group perceptions	C. Stereotypes
	D. Group exclusion
	E. Perceived injustice
	F. Social identity
MC3 Original social networks	G. Familiar communication circles
	H. Online social networks
MC4 Subjective interaction intention	I. Reluctance to communicate
	J. Willingness to communicate
MC5 Objective interaction opportunities	K. Time for contact
	L. Opportunities for contact
MC6 Interaction states	M. No interaction
	N. Occasional conversation
	O. Getting along well
